# Digital Health and the Digital Divide: A Bibliometric Analysis of Access and Equity Among Vulnerable Populations (2020–2025)

**DOI:** 10.1002/puh2.70301

**Published:** 2026-06-16

**Authors:** Sweeta Agrawal, Abayomi O. Agbeyangi

**Affiliations:** ^1^ Rama Devi Women's University Bhubaneswar India; ^2^ Walter Sisulu University East London South Africa

**Keywords:** AI‐driven health infrastructure, COVID‐19, digital divide, digital health, global collaboration, marginalized populations, telehealth

## Abstract

**Background:**

The COVID‐19 pandemic catalyzed global digital health deployment while simultaneously magnifying structural inequities affecting marginalized communities, particularly in low‐ and middle‐income countries (LMICs).

**Method:**

Employing a descriptive bibliometric design, we examined 1250 peer‐reviewed articles indexed in Scopus (2020–July 2025). Utilizing Biblioshiny and VOSviewer, this study delineates publication trajectories, collaborative networks, and principal domains of inquiry within digital health equity research.

**Results:**

Analysis reveals an exponential surge in publications post‐2020, with the United States, United Kingdom, China, and Australia as principal producers. Eight thematic clusters emerged, heavily skewed toward technological infrastructure over equity‐centric research. Although international collaboration is expanding, the literature indicates that scholarship in LMICs remains highly dependent on international partnerships.

**Conclusion:**

This analysis highlights that the global knowledge base regarding digital health inequities remains heavily concentrated in well‐resourced regions. To genuinely advance health equity research, future scholarly efforts must prioritize participatory, context‐specific studies that elevate the visibility of underserved populations.

## Graphical abstract

This bibliometric analysis of 1250 Scopus articles (2020–2025) maps global digital health research through a health equity lens. Using Biblioshiny and VOSviewer, eight research clusters were identified. Knowledge production is concentrated in high‐income countries, whereas low‐ and middle‐income regions remain underrepresented, deepening existing digital health inequities.

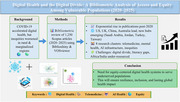

## 1 Introduction

The COVID‐19 pandemic served as a major driver for the sudden adoption of digital health technologies across the world, both in the form of telehealth, mHealth, and eHealth platforms [1]. Although these technologies were designed to address the disruptions to healthcare delivery, their benefits were not evenly distributed. Access to and effective utilization of digital health technologies are still strongly correlated with socio‐economic status and geographical location, as well as preexisting healthcare access [2]. Although mobile connectivity has expanded tremendously, disparities remain; for example, although a large share of adults in developing regions have mobile phones, access to smartphones and reliable internet, which are important for digital healthcare, is uneven.

During the pandemic, digital health progressed from being a complementary service to a vital mode of healthcare delivery. However, for underserved populations, the promise of telehealth was often conflicted with realities, such as lack of internet access, insufficient digital infrastructure, and low levels of digital literacy [3, 4, 5]. These barriers were especially pronounced among older adults, rural populations, and low‐income groups, where preexisting structural disadvantages constrained access to digital services and magnified health inequalities, heightened vulnerability [6, 7, 8, 9].

The concept of the “digital divide,” traditionally used to describe differences in access to information and communication technologies, gained renewed significance during the pandemic, when it became directly linked to health outcomes [10, 11, 12]. Vulnerable groups, such as the elderly, those living in developing countries, and those with limited sources of income or education, were often left out of digital health services that functioned as critical lifelines during lockdowns [13, 14]. For instance, research in Nigeria underlines the lack of adequate digital infrastructure constrained access not only to healthcare but also to health education and services [15]. This divide extends beyond mere access to hardware or internet connectivity; it encompasses disparities in digital literacy, psychosocial barriers, and the availability of robust digital public infrastructure (DPI) [16]. This multidimensional view facilitates a deeper understanding of how technological inequalities translate into disparities in access, use, and wider health equity [13, 14].

These challenges further highlighted the necessity of reconsidering the design and implementation of digital health solutions, especially in ways that account for the social, economic, and technological context of end users [17, 18, 19]. Availability of technology is not enough; digital health systems need to be accessible, usable, and relevant to marginalized populations. This underscores the importance of incorporating equity considerations during both technological innovations and in policy development.

Although prior bibliometric studies have examined digital health, telemedicine, and digital literacy during and after the COVID‐19 period, these analyses have largely focused on growth in publications, change in themes, and citation patterns [20, 21, 22]. Similarly, existing reviews on the digital divide focus on conceptual and technological disparities but do not assess disparities in the production and collaboration of global research in a systematic way [2]. As a result, the intersection of digital health and the digital divide is not sufficiently studied from an equity‐oriented bibliometric perspective. To fill this gap, this study incorporates an explicit equity‐focused lens into its bibliometric analysis. Specifically, it analyzes geographic disparities in research output, highlights imbalances between high‐income and low‐ and middle‐income countries (LMICs), and analyzes collaboration networks shaping global knowledge production. In doing so, the study goes beyond descriptive mapping to interpret how patterns of research activity reflect broader structural inequalities in digital health access and representation.

The major contributions of this study are as follows:

- It analyzes 1250 Scopus‐indexed publications (2020–2025) to map the evolution of digital health research during and after the COVID‐19 pandemic.
- It examines patterns of inequality in global research production, highlighting geographic and institutional concentration.
- It identifies disparities in research representation related to vulnerable populations and underrepresented regions.
- It provides directions for future research on digital health equity, particularly in relation to inclusive and context‐sensitive approaches.

In this regard, the study examines global scholarship by focusing on digital health, with particular attention to disparities in access, adoption, and representation in research. Although the analysis provides a comprehensive bibliometric overview, it is descriptive and does not directly assess the effectiveness of digital health interventions. Rather than evaluating the clinical effectiveness of specific interventions, this study analyzes patterns of knowledge production. However, it provides valuable insights into emerging research trends and structural gaps that can guide future research on digital health equity.

## 2 Methods

### 2.1 Study Design and Rationale

This study employed a descriptive and evaluative bibliometric research design to systematically map the global scholarly discourse surrounding digital health and the digital divide during and post the COVID‐19 pandemic. Unlike traditional systematic reviews, which synthesize empirical findings to answer specific clinical or policy questions, bibliometric analysis uses macro‐level quantitative methods to evaluate research productivity, track thematic evolution, and uncover hidden collaborative networks within a defined scientific domain [23].

To ensure methodological transparency and rigor, the reporting of the data collection and screening phases was guided by the Preferred Reporting Items for Systematic Reviews and Meta‐Analyses (PRISMA) framework. Furthermore, this study adopted a distinct equity‐oriented analytical lens. By examining specific bibliometric indicators, such as the geographic distribution of corresponding authors, single‐country publications (SCP) versus multiple‐country publications (MCP), and institutional affiliations, this design aims to reveal how structural inequalities in global knowledge production mirror the very digital divides the research seeks to address.

### 2.2 Database Selection and Justification

The Scopus database (Elsevier) was selected as the exclusive data source for this retrieval. Scopus was chosen over alternative databases (such as Web of Science or PubMed) due to its superior coverage of interdisciplinary literature, which is essential for a topic that sits at the nexus of clinical medicine, public health, information technology, and the social sciences. Scopus offers an extensive repository of over 90 million records and provides highly structured, standardized metadata (including comprehensive citation tracking, exact affiliation data, and standardized author profiles) that are uniquely optimized for integration with advanced science‐mapping software such as Bibliometrix and VOSviewer [24, 25].

Although relying on a single database might constitute a methodological limitation, potentially introducing a geographic and linguistic bias by overrepresenting English‐language outputs from high‐income countries at the expense of regional LMIC databases, Scopus remains the consensus standard for comprehensive bibliometric mapping and provides a robust, representative sample of the highest impact international literature.

### 2.3 Search Strategy and Data Retrieval

A comprehensive literature retrieval was executed in July 2025. To capture a highly relevant corpus, a comprehensive Boolean search string was constructed using the *TITLE‐ABS‐KEY* (Title, Abstract, Keywords) search field parameter. The query was designed to intersect four primary conceptual domains: digital health modalities, the digital divide, vulnerable demographics, and the pandemic timeline.

The exact query executed was as follows: TITLE–ABS–KEY ((“digital health” OR “telehealth” OR “eHealth” OR “mHealth”) AND (“digital divide” OR “digital inequality” OR “access to technology”) AND (“rural” OR “low‐income” OR “underserved” OR “developing regions” OR “vulnerable populations”) AND (“infrastructure” OR “internet access” OR “digital literacy”) AND (“COVID‐19” OR “pandemic” OR “post‐pandemic”)).

The search was constrained to the publication window of 2020–July 2025 to capture the immediate catalyst of the pandemic and the subsequent stabilization of digital health policies. The initial execution yielded 1956 raw records. A detailed discussion of the methodological concerns inherent to this search strategy and database selection is provided in Section 4.4.

### 2.4 Eligibility Criteria and Screening

**FIGURE 1:**
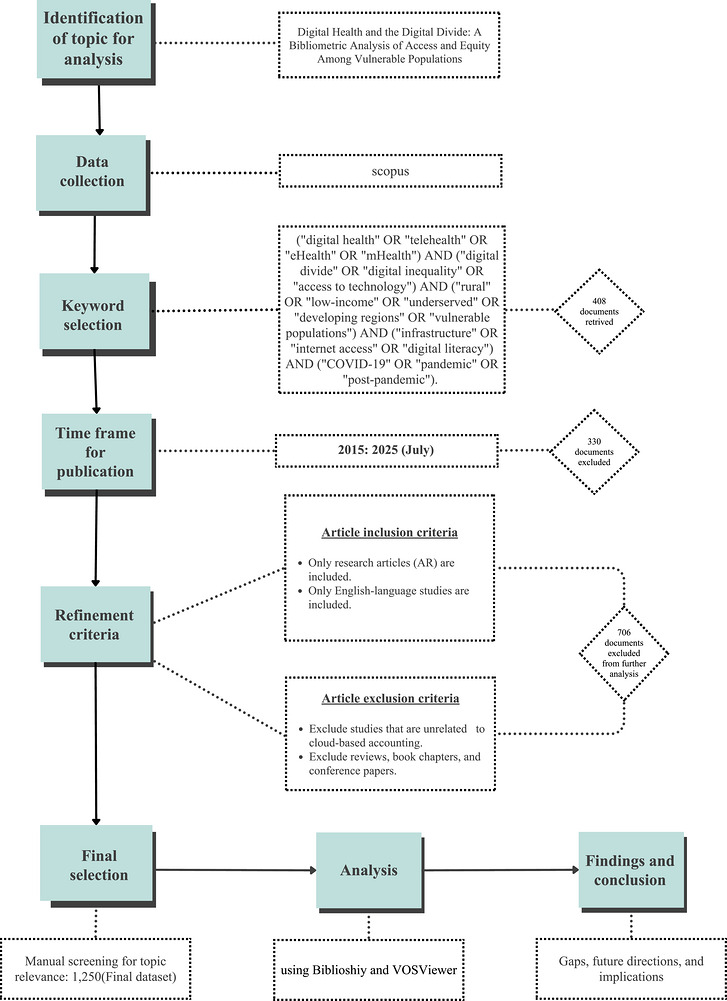
PRISMA model.

To ensure the integrity and empirical focus of the dataset, rigorous inclusion and exclusion criteria were applied. The filtering process proceeded sequentially:

- Document‐type refinement: The dataset was restricted to original, peer‐reviewed research articles. Secondary literature (reviews), editorials, letters, brief notes, and conference proceedings were excluded to focus strictly on primary scholarly production. This reduced the corpus to 1340 articles.
- Publication stage: Only articles in the “final” publication stage were retained, excluding “articles in press” to ensure complete citation metadata, resulting in 1261 records.
- Language: The search was limited to English‐language publications, yielding a final set of 1250 articles.
- Manual screening: Title and abstract screening was conducted independently by authors to identify and eliminate “false positives” (e.g., articles utilizing the keywords metaphorically or focusing on unrelated technological domains). Discrepancies between the authors were resolved through collaborative deliberation until a consensus was reached against the predefined inclusion parameters. The complete attrition process is visualized in the PRISMA flow diagram (Figure 1).

### 2.5 Data Harmonization and Management

Before quantitative mapping, the final dataset underwent a rigorous manual harmonization process in Microsoft Excel. This included keyword consolidation, specifically merging synonymous or varied spellings in Author Keywords (e.g., standardizing “e‐health,” “eHealth,” and “electronic health” into a single node, or merging “COVID‐19” and “SARS‐CoV‐2”). This cleaned matrix was then imported into the analytical software environments to ensure high reliability in subsequent network visualizations.

### 2.6 Analytical Framework and Science Mapping

The analysis was executed in two distinct phases utilizing two complementary software packages:

- Phase 1: Performance Analysis (Biblioshiny): Biblioshiny, the web‐based interface for the R package Bibliometrix, was deployed to evaluate descriptive metadata. This tool extracted annual trajectories of scientific production, source impacts, country‐level collaboration metrics (SCP/MCP ratios), and global citation impacts.
- Phase 2: Science Mapping (VOSviewer): VOSviewer (Version 1.6.20) was utilized to construct the conceptual and intellectual architecture of the field. Two specific network models were generated: a country‐perspective bibliographic coupling network to reveal shared intellectual interests, and a keyword co‐occurrence analysis to identify thematic clusters.

For the VOSviewer mapping parameters, the full‐counting method was used to ensure equal weighting of all relationships, and association‐strength normalization was applied to balance the scale of the network graphs [25]. To maintain network clarity and focus strictly on significant research trends rather than isolated studies, a minimum occurrence threshold of five (5) was established for keywords. Nodes within the resulting visualizations represent entities (countries or concepts), whereas node size corresponds to publication frequency, and link thickness indicates the strength of the relationship.

## 3 Results

### 3.1 Bibliometric Trends, Growth Pattern, and Citation Analysis

The study analyzed bibliometrics from 2020 to July 2025 and found 1250 documents from 590 different sources. Following the documents, the publication trend demonstrated rapid growth, starting with 24 documents in 2020, peaking at 366 in 2024, with 269 recorded in 2025 (partial year; data through July 2025 only—annualized projection: ∼460). The annual growth rate from 2020 to 2025 was 62.15%, indicating that this area is rapidly evolving and is now attracting significant scholarly attention. The annual growth rate was calculated as the percentage increase in the number of publications over the study period, providing an indicator of the pace of research expansion in digital health equity.

The average age of the documents is 1.68 years, indicating that the research is still relatively new. The average document citation is 10.99, although a clear pattern emerges—Documents from 2020 and 2021 garnered more attention, with means of 63.33 and 35.04, respectively. Citation impact was assessed using total citations and average citations per document, providing a measure of research influence and visibility across the field. In contrast, documents from 2024 and 2025 had significantly lower values of 4.32 and 0.98, indicating that they have had little time to gain more attention.

The literature demonstrates a high level of collaboration across the documents, with the vast majority being multi‐authored (mean: 15.3 co‐authors per document). These involved 9606 scholars, with an average of 15.3 co‐authors per document, and 20.8% of the documents were collaborations with other countries. Collaboration rate was defined as the proportion of publications involving authors from multiple countries, reflecting the extent of international research cooperation. This international collaboration is a clear indication of strong interdisciplinary and cross‐border scholarly activity.

**FIGURE 2:**
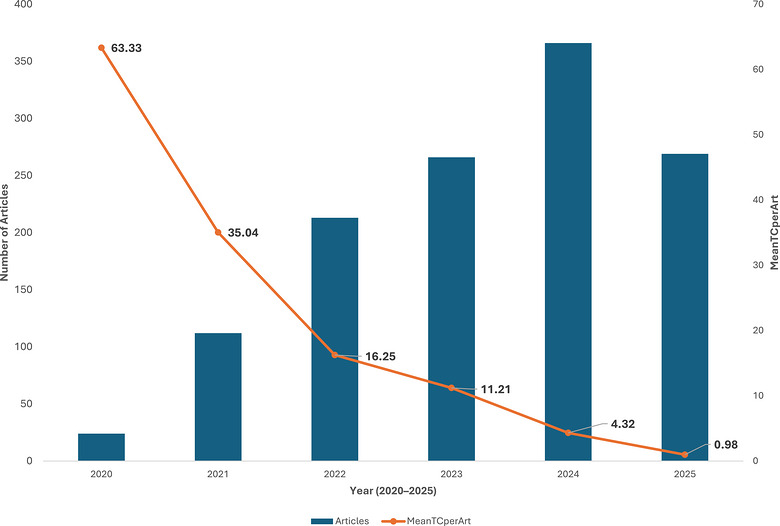
Trends in research output and citation impact.

The thematic diversity within the field is striking, as evidenced by 3784 Keywords Plus and 5973 Author Keywords, reinforcing a wide‐ranging and interconnected field of study supported by 10,038 cited works. Figure 2 presents the number of published articles per year (bars, left axis) and the average number of citations per article (line, right axis) on digital health equity during the period 2020–2025. The figure also illustrates the annual number of published articles (bars, left axis) and the mean citations per article (line, right axis) on digital health equity between 2020 and 2025.

### 3.2 Most Prolific Journals, Author Affiliation, and Country‐Level Contributions

The 1250 publications were distributed across an extensive range of journals, indicating the multidisciplinary nature of digital health research. Of the 1250 publications, 285 appeared in the 10 most prolific journals; percentages in Table 1 are calculated from this subset. As shown in Table 1, the *Journal of Medical Internet Research* was the most prolific source, accounting for 22.11% of the top‐journal output, followed by Digital Health and Frontiers in Public Health. The preponderance of journals devoted to health informatics, public health, and digital medicine suggests that the discipline has a strong grounding in applied health sciences, with an increasing integration of technological and social perspectives. This distribution implies that digital health equity is primarily addressed through clinical and population health lenses rather than purely technological domains.

**TABLE 1:** Top 10 journals in digital health equity research.

Rank	Journal title	Frequency (*n*)	% (*n* = 285)	Subject area
1	*Journal of Medical Internet Research*	63	22.11	Medicine, health informatics
2	*Digital Health*	43	15.09	Medicine, public health, health services
3	*Frontiers in Public Health*	31	10.88	Public health, social sciences
4	*BMC Public Health*	26	9.12	Public health, epidemiology
5	*International Journal of Environmental Research and Public Health*	26	9.12	Public health, environmental sciences
6	*Telemedicine and e‐Health*	25	8.77	Health informatics, medicine
7	*JMIR Formative Research*	20	7.02	Digital health, medicine
8	*Healthcare (Switzerland)*	19	6.67	Medicine, health services
9	*Plos One*	17	5.96	Multidisciplinary science
10	*Sustainability (Switzerland)*	15	5.26	Environmental science, social sciences

*Note:* The table presents the 10 most active journals contributing to digital health equity research from the analyzed dataset (*n* = 1250).

Subject area classification further supports this interdisciplinary trend, with the largest proportion in Medicine (62.96%), followed by Social Sciences (26.00%) and Computer Science (17.12%). Percentages sum to more than 100 as Scopus classifies articles under multiple subject areas; figures reflect the proportion of documents tagged to each domain. The existence of fields like business, psychology, and environmental science shows that digital health equity is becoming more widely recognized as a multifaceted issue with behavioral, organizational, and systemic dimensions. This wide‐ranging disciplinary involvement demonstrates the complexity of digital inequalities in healthcare access and delivery.

At the institutional level, there is a significant concentration of research output among leading academic and medical institutions, as shown in Table 2. The University of California leads with 58 publications (4.64%), followed by UCSF School of Medicine and Mayo Clinic. Notably, most leading contributors are from the United States, with limited representation from LMICs. This pattern suggests that research capacity and institutional leadership for digital health are concentrated in well‐resourced academic environments with the potential to influence the global research agenda and priorities but are limited in low‐ and middle‐income environments.

**TABLE 2:** Leading institutions publishing on digital health and equity issues.

Rank	Institution	Frequency (*n*)	% (*n* = 1250)	Country
1	University of California	58	4.64	United States
2	UCSF School of Medicine	40	3.20	United States
3	Mayo Clinic	36	2.88	United States
4	Harvard Medical School	31	2.48	United States
5	Duke University School of Medicine	25	2.00	United States
6	The University of British Columbia	22	1.76	Canada
7	UAMS College of Medicine	21	1.68	United States
8	University College London	21	1.68	United Kingdom
9	VA Medical Center	21	1.68	United States
10	Johns Hopkins Bloomberg School of Public Health	20	1.60	United States

*Note:* The table lists the top 10 institutions most frequently publishing on digital health and equity issues, based on 1250 analyzed records.

**FIGURE 3:**
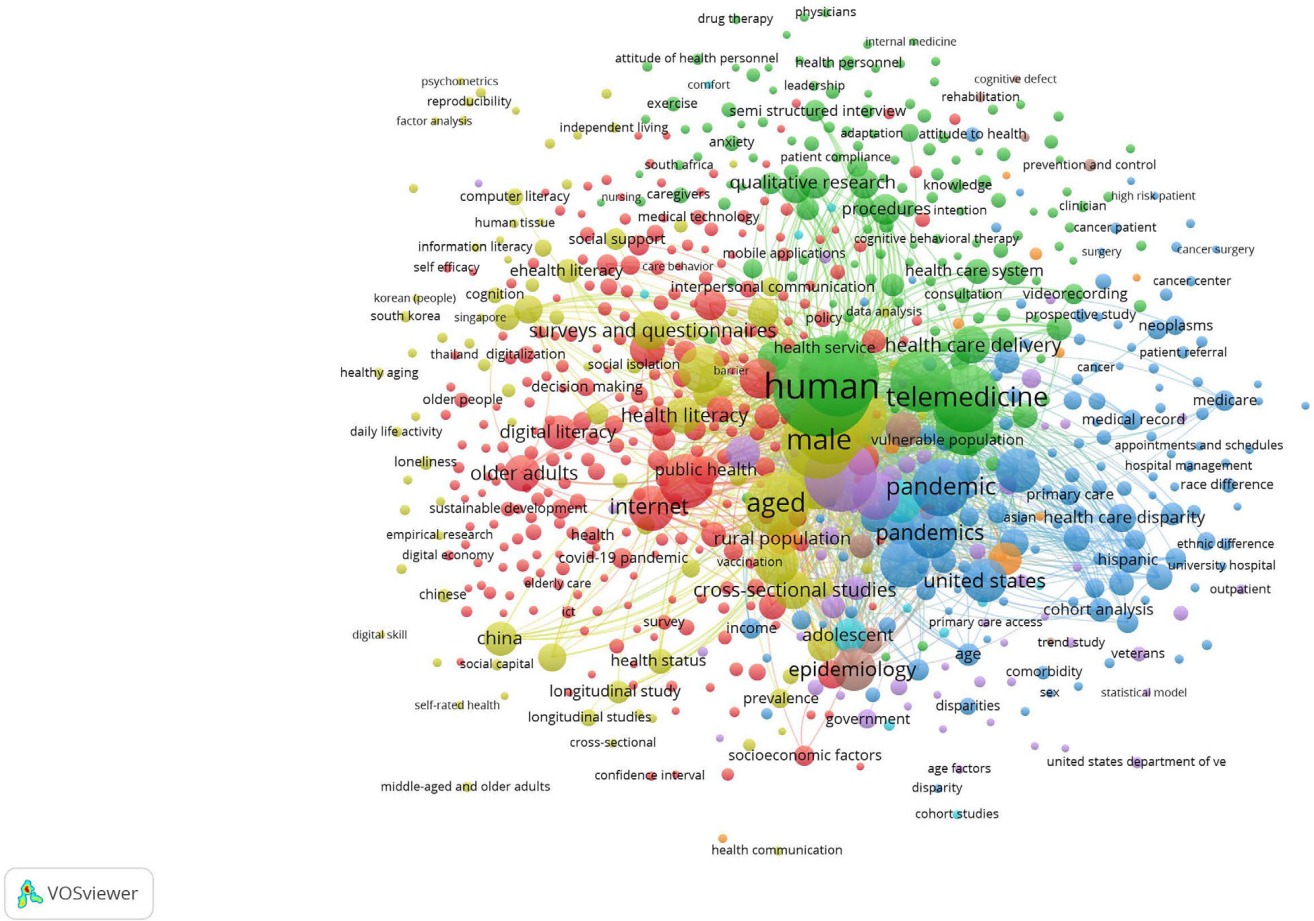
Network map of author keywords highlighting major research hotspots as prominent nodes. The figure presents a bibliometric network map generated using VOSviewer, illustrating co‐occurring author keywords from 1250 retrieved publications. Nodes represent keywords (sized by frequency), and colors indicate eight thematic clusters that emerged as research hotspots.

A similar pattern is seen at the country level. Table 3 presents the top 10 countries by article output. The full bibliographic coupling network across all 50 contributing countries is visualized in Figure 3. As shown in Table 3, the United States leads by a substantial margin, followed by China, Australia, Canada, and the United Kingdom. High‐income countries not only lead in total output but also in independent research capacity, as measured by high SCP counts. For example, the United States shows strong independent leadership (418 SCP out of 425 articles), whereas countries, such as Kenya and Uganda, rely entirely on international collaboration (MCP = 100%), with no SCPs.

**TABLE 3:** Corresponding author countries: comparison of research leadership and collaboration patterns.

Rank	Country	Articles	SCP	MCP	MCP %
1	United States	425	418	7	1.6
2	China	169	128	41	24.3
3	Australia	79	42	37	46.8
4	Canada	77	40	37	48.1
5	United Kingdom	55	45	10	18.2
6	Germany	24	17	7	29.2
7	India	23	15	8	34.8
8	Indonesia	22	20	2	9.1
9	Kenya	3	0	3	100.0
10	Uganda	2	0	2	100.0

Abbreviations: MCP, multiple‐country publications; SCP, single‐country publications.

This contrast underscores a major imbalance in global research leadership. Although high‐income countries are positioned as primary producers of knowledge, LMICs are largely positioned as collaborative participants rather than independent contributors. Such reliance on international collaboration points to shortcomings in local research infrastructure and funding capacity in the field. Consequently, this imbalance may affect whose perspectives are being represented in the literature and how digital health equity challenges are framed and addressed globally. These findings suggest that, despite digital health equity being a global issue, knowledge production remains unevenly distributed, with important implications for inclusivity, representation, and policy relevance in this field.

### 3.3 Emerging Hotspots in Digital Health Equity Research Identified From Author Keywords

**FIGURE 4:**
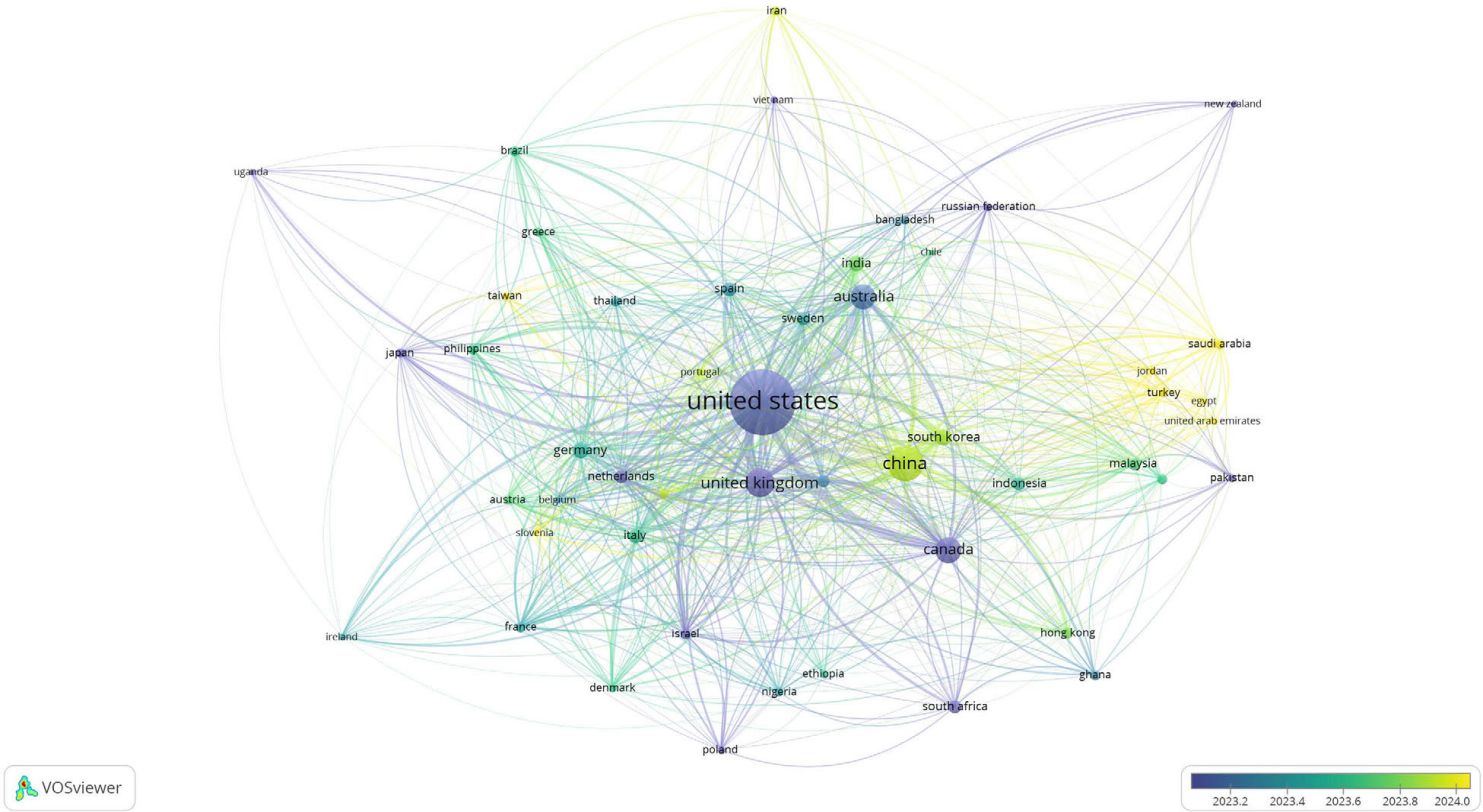
Bibliographic coupling network of countries in digital health equity research. The network visualization, generated using VOSviewer, maps bibliographic coupling among 50 countries. Node size reflects publication volume, whereas link strength indicates the degree of shared citation patterns.

The co‐occurrence analysis of author keywords revealed 741 keywords (minimum occurrence ≥5). These were grouped into eight distinct thematic clusters (Figure 4). These clusters represent the intellectual structure of the field and can be broadly grouped into three major domains: (i) technology‐based research, (ii) clinical and service‐based applications, and (iii) equity‐ and population‐based studies.

As summarized in Table 4, the largest cluster is centered around digital infrastructure, artificial intelligence, and technology adoption. This shows that the field is mainly driven by technological innovation, with a strong focus on tools such as AI, big data, and digital technologies. Closely related clusters include the topics of telehealth, clinical practices, and mental health services, reflecting the acceleration of digital healthcare delivery during the COVID‐19 pandemic.

**TABLE 4:** Thematic clusters and key research hotspots.

Cluster	Theme	Representative keywords	Interpretation
1	Digital Infrastructure & AI	Digital health, AI, big data, privacy, broadband	Technology‐driven research dominates the field
2	Clinical Care & Mental Health	Mental health, telehealth, communication, CBT	Focus on service delivery and patient interaction
3	Health Disparities & Equity	Disparities, race, access, inequality	Core equity–focused research
4	Digital Literacy & Ageing	eHealth literacy, ageing, chronic disease	Focus on older and vulnerable populations
5	Telehealth & Service Access	Teleconsultation, outpatient care, HIV, COPD	Expansion of remote healthcare services
6	Early Life & Digital Access	Children, families, access, cost	Emerging but underexplored area
7	Rural Health & Maternal Care	Rural health, pregnancy, access barriers	Geographic inequalities in access
8	Epidemiology & Pandemic Response	COVID‐19, prevention, rehabilitation	Pandemic‐driven research foundation

Several clusters explicitly address issues of health disparities, vulnerable populations, and access barriers. These include racial and ethnic inequalities research, aging population, rural healthcare accessibility, and early life digital exclusion. However, these equity‐focused clusters are more dispersed and less dominant than those related to technology, which may indicate that issues related to the digital divide are often embedded within broader clinical or technological exploration rather than being central themes.

A notable pattern is the strong representation of mental health and telemedicine across multiple clusters, indicating their centrality in healthcare responses in digital health during the pandemic. At the same time, emerging dimensions, such as early‐life digital access and rural healthcare, are comparatively underexplored, suggesting possible gaps in the literature. It should be noted that there is a clear thematic overlap between clusters, most notably between Cluster 2 (clinical care and mental health) and Cluster 5 (telehealth and service access), both of which address remote service delivery and patient interaction. Similarly, Cluster 3 (health disparities and equity) has conceptual commonalities with Clusters 4 and 7, which also address vulnerable and underserved populations. This degree of overlap is a reflection of the inherently linked nature of digital health equity as a field of research rather than a shortcoming of the clustering approach; the co‐occurrence thresholds were applied in a consistent manner, and the detected degree of convergence across clusters strengthens the centrality of equity and access as cross‐cutting concerns across the literature.

Overall, the thematic structure suggests that although digital health research has advanced rapidly in response to the COVID‐19 pandemic, the focus remains on technological solutions and service delivery. Issues related to equity, access, and structural disparities, while present, are comparatively less focused on. This imbalance points to an important gap between technological innovation and effective, equitable implementation, underscoring the need for more targeted research on underserved populations and digital health equity.

### 3.4 Bibliographic Coupling Network: Country Perspective

The bibliographic coupling analysis of the 50 countries shows five distinct clusters based on similar citation patterns and knowledge bases (Figure 4). These clusters reflect intellectual connections among countries through the literature they cite; they offer insight into the structure of global knowledge production in digital health equity research.

The network reveals a well‐defined core–periphery structure. Several countries, such as the United States, the United Kingdom, China, and Australia, serve as central nodes, indicating strong integration within the global research system and shaping the field's knowledge base. Their high connectivity indicates that they are likely to act as primary knowledge producers, shaping research direction and providing foundational literature widely cited in other countries. In contrast, a number of emerging contributors, including Saudi Arabia, Jordan, Turkey, Egypt, Taiwan, and Slovenia, are located toward the semi‐periphery of the network. The overlay visualization suggests that these countries are increasingly citing the same core literature as dominant nations, suggesting a move toward static research themes. This pattern reflects increasing involvement in the global research landscape but also dependence on existing knowledge frameworks rather than the pursuit of independent research trajectories.

These emerging countries also seem to play a role as regional connectors, facilitating knowledge exchange across regions such as the Middle East, Europe, and Asia. Their growing integration into the network might be explained by increased research investments, international collaborations, and work on interdisciplinary topics such as digital technologies for health, sustainability, and AI governance.

However, the network structure also shows an imbalance in the influence of knowledge worldwide. Although the high‐income countries are at the center and play influential roles, many LMICs are peripheral or weakly connected in the network. This indicates that their contributions may have low visibility and relevance to the overall research agenda. Such imbalances in knowledge centrality reinforce inequalities in current research systems worldwide and may limit the diversity of perspectives and voices in digital health equity research.

Overall, the bibliographic coupling network shows that global knowledge production is highly interconnected but unevenly distributed in this field, with a focus of influence between a small group of dominant countries. This has important implications for the inclusivity and representativeness of research on digital health equity.

### 3.5 Top 10 Most Cited Documents

Table 5 presents the most frequently cited publications on digital health equity, along with influential publications that shaped the research landscape during and after the COVID‐19 pandemic. To ensure a more balanced assessment of impact, citation performance was determined not only by total citations, but also by citations per year and normalized citation scores, and could therefore be compared for earlier and more recent publications.

**TABLE 5:** Most cited articles on digital health and equity issues among vulnerable populations during and after COVID‐19.

Title	Year	Source title	Total citations	Citations per year	Normalized citation score	DOI
Digital Transformation in Healthcare: Technology Acceptance and Its Applications	2023	*International Journal of Environmental Research and Public Health*	394	98.50	35.13	10.3390/ijerph20043407
Bridging Digital Divides: A Literature Review and Research Agenda for Information Systems Research	2023	*Information Systems Frontiers*	246	61.50	21.94	10.1007/s10796‐020‐10096‐3
When Going Digital Becomes a Necessity: Ensuring Older Adults’ Needs for Information, Services, and Social Inclusion During COVID‐19	2020	*Journal of Aging & Social Policy*	239	34.14	3.77	10.1080/08959420.2020.1771237
Impact of COVID‐19 Related Social Support Service Closures on People With Dementia and Unpaid Carers: A Qualitative Study	2021	*Aging & Mental Health*	204	34.00	5.82	10.1080/13607863.2020.1822292
The Effects of COVID‐19 Among the Elderly Population: A Case for Closing the Digital Divide	2020	*Frontiers in Psychiatry*	192	27.43	3.03	10.3389/fpsyt.2020.577427
Impact of COVID‐19 on the Digital Divide: A Rapid Review	2021	*BMJ Open*	177	29.50	5.05	10.1136/bmjopen‐2021‐053440
Ten Questions Concerning Age‐Friendly Cities and Communities and the Built Environment	2021	*Building and Environment*	175	29.17	4.99	10.1016/j.buildenv.2021.107922
Who Gets to Learn in a Pandemic? Exploring the Digital Divide in Remote Learning During the COVID‐19 Pandemic in Nigeria	2021	*International Journal of Educational Research Open*	171	28.50	4.88	10.1016/j.ijedro.2020.100022
The Need for a Mental Health Technology Revolution in the COVID‐19 Pandemic	2020	*Frontiers in Psychiatry*	171	24.43	2.70	10.3389/fpsyt.2020.00523
Telehealth Use Among Older Adults During COVID‐19: Associations With Sociodemographic and Health Characteristics, Technology Device Ownership, and Technology Learning	2022	*Journal of Applied Gerontology*	142	28.40	8.74	10.1177/07334648211047347

*Note:* The table presents the top 10 most cited articles (2020–2025) addressing digital health and equity among vulnerable groups, particularly in the context of the COVID‐19 pandemic.

A definite theme emerges from these highly cited studies. The literature is heavily focused on digital transformation in healthcare, the digital divide, and the role of technology in healthcare access for vulnerable populations. Several influential publications focus on older people, emphasizing the importance of digital exclusion in limiting access to essential health and social services during the pandemic. Mental health is also a dominant theme, indicating the broad use of digital platforms to help support psychological health during periods of lockdown and limited access to healthcare.

In addition, several studies point to disparities in access to telehealth, digital literacy, and remote service delivery [18]. For example, research on dementia care and remote learning environments highlights the disproportionate impact of service disruptions and connectivity limitations on vulnerable groups. Geographic inequalities are also evident, especially in studies conducted in low‐resource settings, where infrastructural barriers limit access to digital health and education services.

However, citation numbers should generally be interpreted with temporal context in mind; however, the highest cited paper in Table 5 is from 2023, indicating that citation velocity—rather than publication age alone—can determine a paper's relative impact over short periods. This bias toward the start of the pandemic could lead to the overrepresentation of pre‐pandemic research in overall citation rankings. When these are adjusted for citations per year, more recent publications, in particular those from 2022 and 2023, show strong, rapidly growing influence, suggesting a shift from a sustained, evolving research theme to an immediate response to the pandemic.

Another key finding is the clustering of highly cited research in high‐income country contexts, indicating that influential research in this area remains heavily biased toward well‐resourced settings. Although some research questions related to LMICs are still comparatively less cited among the most cited studies, this reflects broader inequalities in global research visibility and impact.

Overall, the most cited literature emphasizes technological adoption and pandemic‐driven healthcare challenges, with growing, but still limited, attention to structural inequalities and global inclusivity. This pattern highlights the need for more balanced and context‐sensitive research that better represents diverse populations and settings in digital health equity.

## 4 Discussion

### 4.1 Key Findings

This bibliometric analysis maps the global research landscape on digital health disparities affecting marginalized populations during and after the coronavirus (COVID‐19), highlighting authorship, collaboration, and themes. It maps the knowledge production rather than considering the effectiveness of interventions or the outcomes of policies; in this case, the conclusions are proportionate to the scope. The findings nonetheless have significance in academia, policy, and health service delivery because digital health may reinforce or diminish inequalities depending on contextual realities—such as technology availability for rural populations—or platform accessibility for migrant populations [26, 27, 28, 29, 30]. The analysis explores equity in terms of publication volume, citation impact, and collaboration level using measurable bibliometric indicators, such as SCP/MCP ratios, normalized citation scores, and network centrality measures (Sections 3.2 and 3.4). Regional socio‐economic conditions, political commitments, and cultural factors remain strong determinants of both research output and equitable access to health in Australia, the United States, Europe, and emerging economies [31, 32, 33, 34].

The 1250 publications identified 5 major clusters of countries, resulting in a core–periphery configuration in global knowledge production (Section 3.4), with the United States, United Kingdom, China, and Australia as central nodes. Their dominance in publication volume and network centrality suggests an alignment with greater resources, strong public–private partnerships, established funding models, and early digital health policies [35]. However, it is crucial to note that these bibliometric indicators, such as bibliographic coupling and SCP/MCP ratios, strictly measure citation, and collaboration structures. Although they highlight unequal patterns of research production and publication visibility, they cannot be utilized as direct proxies to evaluate national digital health policy strength, funding capacity, institutional performance, or real‐world health‐system effectiveness. Leading institutions, including the University of California, Harvard, and Oxford, reflect global leadership trends observed across research areas [33, 34]. Less than 10% of publications were from low‐ and middle‐income regions, highlighting ongoing imbalances in global research representation. Kenya and Uganda had 100% MCP rates, indicating high reliance on international partnerships and a lack of independent research capacity. Studies from Nigeria further emphasize that the lack of digital infrastructure limits access to healthcare, health education, and remote services, yet such contexts are among the least cited [35, 36, 37].

Collaboration patterns confirm a high level of cross‐national authorship, especially among the United States, United Kingdom, Australia, China, and Canada, with the major hubs serving as gateways for the flow of knowledge worldwide [38]. Emerging Contributors: Saudi Arabia, Jordan, Turkey, Egypt, Slovenia, and Taiwan have shown a fast increase in output reflecting the wider adoption of telehealth in rural and underserved settings [28, 39]. Several act as “bridging” countries linking Asian and Middle Eastern networks into global health discourse [40], spurred by investments, such as Saudi Arabia's National Digital Health Strategy and Jordan's European telehealth consortia alliances. Singapore and Saudi Arabia similarly act as intercontinental connectors, enabling cross‐regional learning in niche fields, such as cancer care, nephrology, and community mental health. However, emerging nations depend primarily on Western partnerships, raising concerns about their research autonomy and local capacity building (high MCP ratios and peripheral positioning, as shown in Sections 3.2 and 3.4).

Keyword co‐occurrence analysis identified eight clusters across three domains (Table 4): technology‐driven research (Cluster 1: digital infrastructure and AI; Cluster 8: epidemiology and pandemic response), clinical and service‐oriented applications (Cluster 2: clinical care and mental health; Cluster 5: telehealth and service access), and equity‐focused research (Cluster 3: health disparities and equity; Cluster 4: digital literacy and aging; Cluster 6: early life and digital access; Cluster 7: rural health and maternal care). Equity‐oriented clusters are still more fragmented and less predominant than technology‐oriented themes, with early‐life digital access and rural maternal health posing vital underexplored gaps. Digital platform use also raises ethical dilemmas regarding data security and patient confidentiality, especially in areas with underdeveloped digital governance frameworks.

The rise in the output of emerging nations is a reflection of the catalytic role of the pandemic (COVID‐19), alignment with Sustainable Development Goals, and context‐specific needs, such as disaster preparedness, access to digital devices in rural areas, and multi‐language communication [38, 39, 40]. The pandemic also led to some useful care innovations, remote consulting, mobile health units, and localization of platforms that bridged immediate service gaps. Research from 2022 onwards increasingly focuses on rural, marginalized, and non‐English‐speaking populations using user‐centered, co‐design, and culturally competent approaches reflecting rapid reviews prioritizing trust‐building and user experience to adopt success [17].

Although previous bibliometric reviews of digital health during the COVID‐19 era have primarily charted publication growth, clinical thematic shifts, and basic citation patterns [20, 21, 22], this study adds distinct descriptive value by integrating a core–periphery framework to quantify structural inequalities within the knowledge base itself. By mapping bibliographic coupling networks, we illustrate that the global discourse is not merely expanding but remains highly centralized around a few high‐income nations, thereby perpetuating an imbalance in global research representation. Furthermore, our equity‐focused keyword cluster analysis differentiates this work from standard technological mappings by explicitly revealing a thematic asymmetry: innovation‐driven infrastructure clusters vastly overshadow localized structural equity and access concerns. Consequently, this analysis moves beyond merely describing what digital health technologies are being published to offer a critical, macro‐level perspective on whose contextual realities and systemic priorities are disproportionately shaping the future of global digital health equity research.

### 4.2 Theoretical and Methodological Implications

The application of a core–periphery theoretical framework to the bibliographic coupling network (Section 3.4) provides a structured lens for interpreting patterns of knowledge concentration and dependency in global digital health research. The presented framework shows that only a few high‐income countries are located at the center of global knowledge generation (mainly the United States, United Kingdom, China, and Australia), whereas most countries, especially LMICs, are on the periphery and often rely on partnerships with core countries to gain publication visibility and citation impact. This structural asymmetry is not just descriptive but reflects and potentially reinforces the very digital divides the research ostensibly aims to address. The focus of high‐citation studies in high‐income country settings further underscores the similarity between research influence and existing inequalities worldwide.

Methodologically, incorporating SCP/MCP ratios, normalized citation scores, and network centrality measures into the bibliometric toolkit is a step toward operationalizing equity as a measurable rather than merely a narrative issue. The eight thematic clusters identified through keyword co‐occurrence further illustrate what bibliometric tools can help discover about structural imbalances in research emphasis, showing, for example, that clusters related to technology development are preponderant over those related to equity. Future bibliometric studies in this domain would benefit from the addition of this analytical repertoire, for example, by incorporating LMIC‐to‐HIC citation flow ratios or collaboration asymmetry indices to enable more granular assessments of knowledge power dynamics.

### 4.3 Recommendations and Future Prospects

This bibliometric analysis highlights structural patterns and knowledge gaps that, although they do not permit direct evaluation of intervention outcomes, suggest several directions for future research and policy consideration. These observations are based on patterns in the bibliometric data and are presented as evidence‐informed directions rather than prescriptive policy mandates. There are five strategic directions put forward:

- Strengthen research capacity in LMICs: Concentration of output in high‐income countries and full collaborative dependency of LMIC contributors such as Kenya and Uganda (MCP = 100%; Section 3.2) suggests that global partnerships must go beyond data sharing. Building the necessary infrastructure, training researchers, and open‐access directories are necessary for the marginalized regions to be able to generate, document, and disseminate original knowledge on digital health equity—including in sub‐Saharan Africa and South Asia [41].
- Promote context‐sensitive and culturally adapted digital interventions: Bibliometric evidence showing underrepresentation of rural, language‐minority, and low‐literacy populations in current research, particularly the comparatively low publication volume of Clusters 6 and 7, suggests that future work should focus on local socioeconomic and linguistic dynamics [42]. Co‐designing digital interventions in collaboration with communities, especially in rural areas or with aging or low‐literacy populations, is likely to increase adoption and sustainability [43].
- Expand multidisciplinary collaboration and policy integration: Thematic cluster analysis shows that these equity‐focused clusters (Clusters 3, 4, 6, and 7) are fragmented compared to technology‐focused domains. Broader interdisciplinary frameworks that combine public health, the social sciences, behavioral sciences, and informatics may provide more comprehensive insights and inform national digital health strategies and the WHO's Digital Health Action Plan.
- Leverage artificial intelligence and big data responsibly: The predominance of Cluster 1 (digital infrastructure and AI) in the keyword co‐occurrence network suggests the accelerated adoption of AI in digital health research. Bibliometric patterns do not alone determine whether current AI applications are equitably designed or governed; however, they do signal a need for equity and ethics considerations to accompany technological innovation, especially with respect to data access disparities between high‐ and low‐resource settings.
- Encourage longitudinal and impact‐oriented research: Most of the publications identified in this review were cross‐sectional and descriptive, suggesting a gap in the lack of longitudinal evaluations of digital health equity. Future studies should include evaluations of health outcomes, policy impacts, and user experiences over time to make a more significant advance in understanding digital transformation beyond publication and citation mapping and to assess whether the real‐world consequences of digital transformation for health equity are further measured in the post‐pandemic recovery and future global crises.

These directions collectively point toward digital health research ecosystems that are equity‐driven, values‐based, and participatory in design, though realizing this vision will require sustained commitment from both research institutions and policy actors.

More so, as observed in recent literature on non‐communicable diseases (NCDs), whereas telemedicine can facilitate patient‐centered care, the psychosocial impacts of the digital divide remain profound [44, 45]. Addressing these disparities requires a paradigm shift toward promoting “digital well‐being” that ensures technological engagement supports complete physical and mental health without introducing new burdens [46]. This necessitates the development of robust DPI. Frameworks, like the India Stack [47], demonstrate how open, interoperable digital systems can scale access to essential services. However, future digital health deployments must be anchored in robust governance frameworks and ethical AI practices to protect data privacy and prevent bias.

### 4.4 Limitations

Some limitations should be recognized. First, the exclusivity of the Scopus database may introduce geographical and language biases, as publications indexed in Web of Science, PubMed, or regional databases, especially from low‐income countries or languages other than English, may have been excluded, thereby contributing to the overrepresentation of high‐income country research. Future research should triangulate between various databases. Second, bibliometric analysis reflects patterns of knowledge production, not the quality, reach, or results of interventions, and thus represents an inherent gap between bibliometric findings and real‐world application. Third, the Boolean search strategy, although intended to be precise, may have excluded relevant studies that used alternative terminology for digital health equity constructs; no formal sensitivity analysis was performed, and this is recommended for future reviews. Fourth, the potential for citation‐based measures to favor older publications and those from high‐income countries, due to longer accumulation periods and larger readership networks, is partially mitigated by Table 5, which presents citations per year and normalized scores; however, there is still some temporal and geographic skew. Finally, VOSviewer clustering parameters, such as a minimum co‐occurrence threshold of 5 and an association strength normalization method, can affect thematic cluster structures; therefore, the eight identified clusters should not be regarded as taxonomies but rather as indicative of dominant patterns.

## 5 Conclusion

This bibliometric analysis, using Biblioshiny and VOSviewer, mapped global research on digital health disparities among marginalized populations during and after the COVID‐19 pandemic. The findings reveal three major domains: technological, clinical, and equity‐focused research, highlighting a clear imbalance. Although technology‐driven and clinical studies dominate, equity‐focused areas, such as early‐life digital exclusion and rural maternal health, remain underexplored, indicating a persistent gap between innovation and inclusive implementation. Global research output is led by the United States, United Kingdom, China, and Australia, with a strong core–periphery structure. Contributions from LMICs remain dependent on international collaborations, though emerging countries are beginning to bridge regional and interdisciplinary networks. The findings highlight both the potential to widen digital health inequities worldwide and the potential to implement structurally aware, equity‐based approaches to promote health equity, policy innovation, and contextually relevant practice.

Importantly, although these bibliometric data cannot serve as direct evidence of real‐world inequalities in digital health access, service quality, or clinical health outcomes, they provide clear evidence of structural disparities in how digital health knowledge is produced, collaborated on, and disseminated globally. To ensure the research agenda does not inadvertently mirror the digital divides it seeks to study, future scholarship must prioritize building independent research capacity in resource‐constrained settings. Achieving true “digital well‐being” for vulnerable populations will require the development of resilient DPI, ethical AI governance, and participatory research designs that prioritize the socio‐economic realities of the end‐users.

## Author Contributions

*Conceptualization:* Sweeta Agrawal and Abayomi O. Agbeyangi. *Supervision:* Abayomi O. Agbeyangi. *Data curation and formal analysis:* Sweeta Agrawal and Abayomi O. Agbeyangi. *Original draft writing:* Sweeta Agrawal. *Software:* Sweeta Agrawal. *Writing – review and editing:* Sweeta Agrawal and Abayomi O. Agbeyangi.

## Funding

The authors have nothing to report.

## Ethics Statement

The authors have nothing to report.

## Consent

The authors have nothing to report.

## Conflicts of Interest

The authors declare no conflicts of interest.

## Data Availability

The processed data that support the findings of this study are available from the corresponding author upon reasonable request.
